# Assessment of modified chitosan composite in acidic reservoirs through pilot and field-scale simulation studies

**DOI:** 10.1038/s41598-024-60559-9

**Published:** 2024-05-09

**Authors:** Hamid Khattab, Ahmed A. Gawish, Sayed Gomaa, Abdelnaser Hamdy, A. N. El-hoshoudy

**Affiliations:** 1 Petroleum Engineering Department, Faculty of Petroleum & Mining Engineering, Suez University, Cairo, Egypt; 2 Mining and Petroleum Engineering Department, Faculty of Engineering, Al-Azhar University, Cairo, Egypt; 3 Department of Petroleum Engineering, Faculty of Engineering & Technology, Future University in Egypt, New Cairo, Egypt; 4 Reservoir Engineering Department, Khalda Petroleum Company, Cairo, Egypt; 5 PVT lab, Production Department, Egyptian Petroleum Research Institute, Cairo, 11727 Egypt; 6 PVT service center, Egyptian Petroleum Research Institute, Cairo, 11727 Egypt

**Keywords:** Biopolymers, Core flooding, Chitosan, Rheological criteria, Simulation studies, Chemistry, Engineering, Materials science, Mathematics and computing

## Abstract

Chemical flooding through biopolymers acquires higher attention, especially in acidic reservoirs. This research focuses on the application of biopolymers in chemical flooding for enhanced oil recovery in acidic reservoirs, with a particular emphasis on modified chitosan. The modification process involved combining chitosan with vinyl/silane monomers via emulsion polymerization, followed by an assessment of its rheological behavior under simulated reservoir conditions, including salinity, temperature, pressure, and medium pH. Laboratory-scale flooding experiments were carried out using both the original and modified chitosan at conditions of 2200 psi, 135,000 ppm salinity, and 196° temperature. The study evaluated the impact of pressure on the rheological properties of both chitosan forms, finding that the modified composite was better suited to acidic environments, showing enhanced resistance to pressure effects with a significant increase in viscosity and an 11% improvement in oil recovery over the 5% achieved with the unmodified chitosan. Advanced modeling and simulation techniques, particularly using the tNavigator Simulator on the Bahariya formations in the Western Desert, were employed to further understand the polymer solution dynamics in reservoir contexts and to predict key petroleum engineering metrics. The simulation results underscored the effectiveness of the chitosan composite in increasing oil recovery rates, with the composite outperforming both its native counterpart and traditional water flooding, achieving a recovery factor of 48%, compared to 39% and 37% for native chitosan and water flooding, thereby demonstrating the potential benefits of chitosan composites in enhancing oil recovery operations.

## Introduction

**Figure 1:**
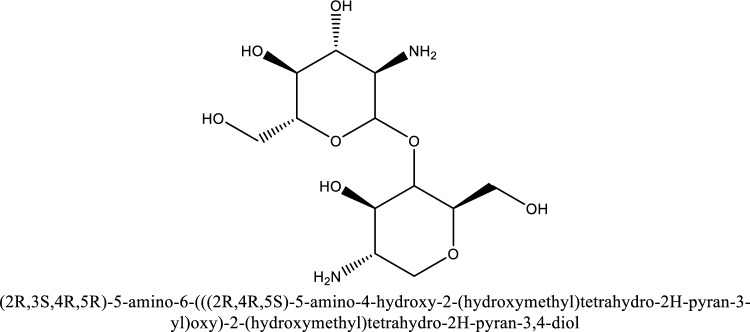
Chitosan chemical structure.

Chitosan is a linear cationic crystalline polysaccharide that is insoluble in pure water but dissolves in pure acetic acid and is produced from the N-deacetylation of chitin^1,2^, which is primarily abundant in the shells of crustaceans like shrimp and crabs^3,4^. Owing to the protonation of the free amino (–NH_3_) groups in chitosan molecular structure, it can be dissolved in aqueous concentrations of organic acids like acetic acid at pH < 6.2^1,3,5^. Chitosan solutions exhibit a reduced viscosity by pH increasing^6^. Chitosan is a versatile bioproduct and is applied for many applications in the petroleum industry, including enhanced oil recovery (EOR), refining, drilling fluid, water treatment, oil spill clean-up, and wellbore treatment. EOR techniques are classified into four categories^7,8^, thermal methods like steam flooding, chemical methods like polymer flooding, miscible methods like CO_2_ flooding, and microbial methods. Polymer injection suffers from significant limitations at hard reservoir conditions of pressure, high temperatures, and extreme salinity^9^. In addition, these polymers are expensive, and not eco-friendly^10^. To overcome these limitations, biopolymers, including chitosan, have been explored. Chitosan boosts the viscosity of displacing fluids, is resistant to mechanical degradation, is non-toxic, inexpensive, and can withstand high temperatures and high salinity^11,12^. Figure 1 is a schematic representation of the chemical structure of chitosan.

Many studies have been conducted to explore different aspects of chitosan's application in EOR, such as its ability to reduce IFT, alter wettability, and increase viscosity. Wang and Xu^13^ investigate the impact of deacetylation on the viscosity and flow characteristics of concentrated chitosan solutions in aquatic media. They discovered that the addition of salt reduces the viscosities and non-Newtonian flow behavior of the chitosan solutions while leaving their flow activation energies^13^. El-hafian et al.^14^ investigated the temperature, shearing time, concentration, and storage time effect on the dynamic viscosity, rheological criteria, and shear stress vs. shear rate of chitosan dissolved in a mildly acidic solution. The results show that the shear thinning performance is very pronounced in the temperature range from 20 to 50 °C^14^. Martinez-Ruvalcaba et al.^15^ screened the effect of the salt and temperature on the chitosan solutions rheology. They reported that the solution viscosity diminished with increasing temperature and salinity^15^. Ghriga et al.^16^ explored gelation in partially hydrolyzed polyacrylamide-polyethylenimine blends. They found that gelation time decreased with higher concentrations, molecular weights, and temperatures at 80 °C and 3.4 g/L TDS, while it increased with hydrolysis degree. Lebouachera et al.^17^ investigated how polystyrene microspheres enhance hydrolyzed polyacrylamide. They analyzed the impact of microsphere size, concentration, and temperature on zero-shear viscosity, with size and temperature proving the most significant. The optimal zero-shear viscosity at 20 °C was achieved with a 50 ppm concentration of 1000 nm microspheres. Lian^18^ prepared CoFe_2_O_4_/chitosan nanoparticles that exhibit good salt resistance and can reduce the IFT between crude oil/water to ultra-lower values in the Shengli oilfield without other additives, indicating their potential for practical applications^18^. Ghriga et al.^19^ provided a comprehensive review of various polymer/polyethyleneimine (PEI) gels, including PAtBA, PAM, PHPA, HAP, AM/AMPS copolymer, and AM/AMPS/N, N-DMA terpolymer, along with recent advancements and successful field applications. Additionally, they investigated the impact of salinity, polymer, and crosslinker concentrations, as well as temperature, on the thermal gelation kinetics of PHPA/PEI gels to mitigate undesired fluid production. Gelation time exhibited a nonlinear relationship with salinity, temperature, and PHPA concentration, as outlined by a mathematical model. Ghriga et al.^20^ investigated how salinity, polymer, and crosslinker concentrations and temperature affected the thermal gelation kinetics of partially hydrolyzed polyacrylamide (PHPA)/polyethyleneimine (PEI) gels for reducing unwanted fluid production. Gelation time showed nonlinear dependence on salinity, temperature, and PHPA concentration as described by a mathematical model. Boublia et al.^21^ highlights the superior performance of graphene-based PANI gas sensors in terms of sensitivity, energy efficiency, and cost-effectiveness at ambient temperatures. These nanocomposites exhibit enhanced responsiveness, durability, and diverse detection capabilities in sensor devices where, the development and potential industrialization of PANI/graphene-based nanomaterials, has an overgrowing interest. Boublia et al.^22^ discuss the utility of response surface methodology (RSM) in optimizing material characteristics in processes such as composites, blends, and polymer membranes. Furthermore, the manuscript thoroughly explains the theoretical foundations and practical implications of RSM, including comparisons with other optimization techniques like artificial neural networks. Wan-Fen Pu et al.^23^ evaluate the chitosan solution properties after grafting with acrylamide, acrylic acid, and 2-acrylamide-dodecyl sulfonate. The solution exhibited viscoelastic behavior and was implemented in oil recovery through core flooding experiments. Hosein Rezvani et al.^24^ synthesized Fe_3_O_4_/Chitosan nanocomposites and investigated their potential for enhanced oil recovery (EOR) operations. The Fe_3_O_4_/Chitosan exhibits good stability in seawater during dynamic experiments and reduces the IFT and contact angle between seawater and crude oil. The flooding experiments showed an increase of 10.8% in oil recovery compared to seawater injection^24^. Lai^25^ developed a branched-modified chitosan polymer (HPDCS) that displayed superior shear resistance and thickening properties compared to HPAM. Also, in the sand-packed tube displacement experiment, HPDCS demonstrated higher oil recovery potential than HPAM^25^. This behavior can be attributed to HPDCS's capacity to preserve its structural integrity and functionality despite the shearing forces encountered during the oil recovery process, while also enhancing the viscosity of the displacement fluid. Qingyuan Chen^26^ synthesized a new chitosan-modified hyperbranched polymer (HPDACS) for EOR applications. HPDACS improved the recovery factor by 19.20%, higher than those of HPAM and HPDA, indicating its great potential for oil displacement^26^. Jie Yu^27^ developed a modified chitosan functional hydrophobic associative polymer CS-*g*-DLMB/AM/AA using a twin-tail monomer (DLMB), AM, and AA to graft/modify malleated chitosan. The modified chitosan displays superior rheological properties. In core flooding experiments, CS-g-AM/AA & CS-g-DLMB/AM/AA achieve oil displacement higher than that of HPAM. Furthermore, CS-g-DLMB/AM/AA with a twin-tail structure performed oil displacement better than CS-g-AM/AA without a twin-tail structure^27^. Lebouachera et al.^28^ investigated the rheological characteristics of polymer-particle composite (PPC) solutions. They observed that PPC thickening exhibited a linear increase in surface functionality for confinement levels below 10, reflecting polymer-particle interactions. Adsorption was quantified in dilute solutions using zero-shear capillary viscosity in a microfluidic device. Conversely, thinning was observed at confinement levels above 10, which was associated with studies on the effect of salt. Tao Liu^29^ prepared a copolymer (CS-g-AM/AA/NIDA) with good thermal stability, increased viscosity, excellent shear and temperature resistance, and salt resistance, leading to 8.08% increased oil recovery in core flooding experiments^29^. Qingyuan Chen^30^ introduced a chitosan-modified hyperbranched polymer (HPDACS) and assessed its biodegradability. The results showed that the polymer had good biodegradability in sewage-containing bacteria surpassing that of polyacrylamide HPAM and dendritic polymer HPDA^30^. On the other hand, combining the nanoparticle and surfactant with chitosan enhances its performance. The nanoparticle can intensify the viscosity of the displacing fluid, which in turn enhances the sweeping efficiency^31–33^. The physical mixture of biopolymer, and nanoparticles may be detrimental to the whole process due to the synergistic effect under reservoir conditions leading to flocculation of nanoparticles^34^. Consequently, biopolymer is modified through the grafting of nanoparticles on its structure leading to composite formation to overcome synergistic effect drawbacks^35,36^.

**Figure 2:**
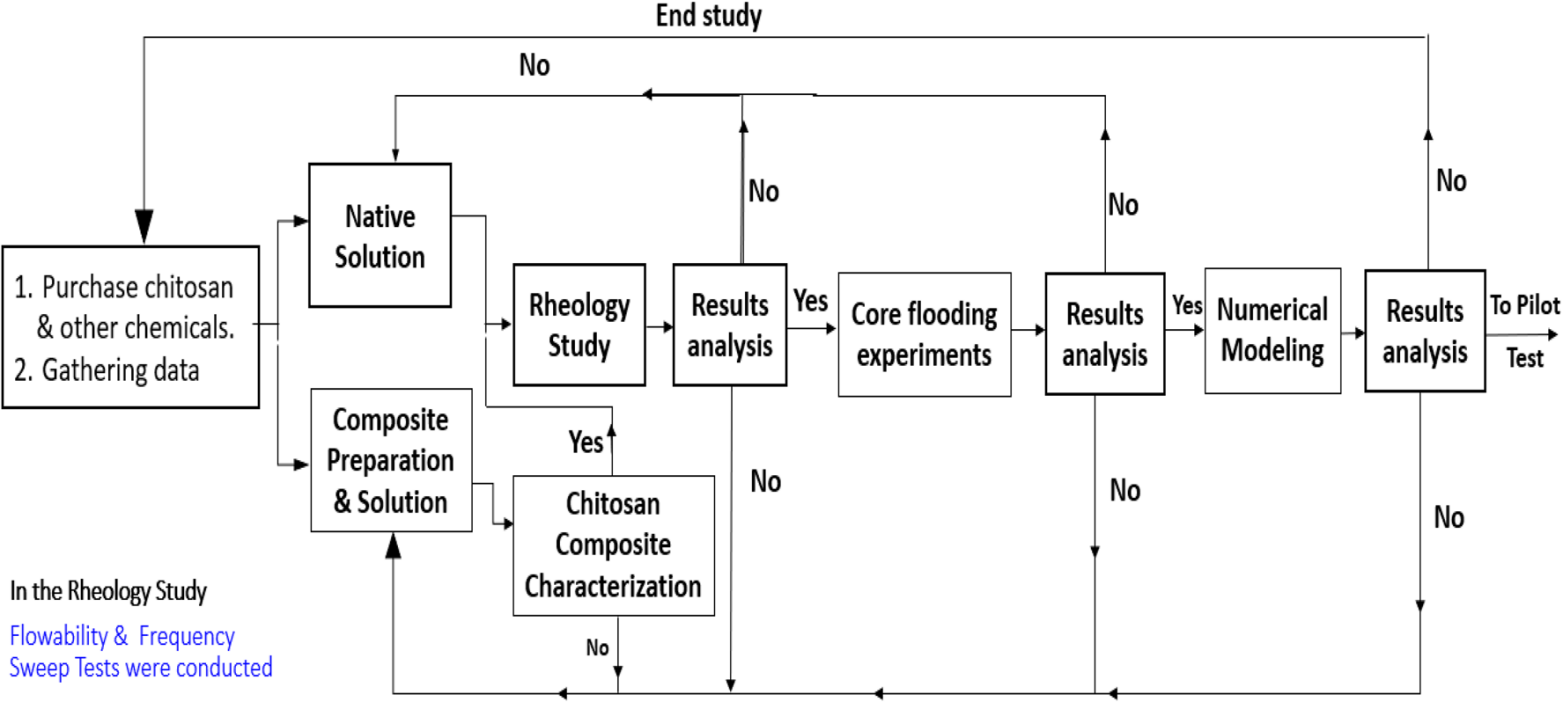
Workflow of the study.

This work discusses the use of chitosan in improved oil recovery to enhance oil recovery in low-permeable acidic reservoirs. The flow work includes the following tasks (1) investigating the effect of temperature, pressure, and salinity on the native and composite chitosan rheological criteria. (2) Both the native and composite chitosan solutions were flooded in a core plug. (3) Conducting a numerical simulation model to detect the change in water viscosity, polymer concentration & variation of mobility ratios with time. Figure 2 displays the study workflow.

The manuscript introduces a comprehensive study on the utilization of chitosan, a biodegradable and environmentally friendly biopolymer derived from the deacetylation of chitin, for enhanced oil recovery (EOR) in the petroleum industry. Highlighting chitosan's unique properties such as solubility in acidic conditions, reduced viscosity with increasing pH, and resistance to extreme conditions like high salinity and temperatures, the manuscript sets the stage for exploring its novel applications in EOR. Prior research has delved into various aspects of chitosan's efficacy in EOR, including viscosity enhancement, wettability alteration, and interfacial tension reduction. This work aims to extend these findings by examining both native and composite chitosan's rheological behaviors under varying reservoir conditions and their impact on oil recovery through core flooding experiments and numerical simulation models. The manuscript's novelty lies in its holistic approach to evaluating chitosan's potential in improving oil recovery in low-permeable acidic reservoirs, promising an eco-friendly alternative to conventional chemical EOR methods.

## Reagents and method

Chitosan powder is supplied from commercial sources. Acrylamide (AM ~ 0.99; CAS 79-06-1); hexadecyltrimethylammonium bromide (CTAB ~ 0.98, CAS 57-09-0); Triethoxyvinylsilane (TEV ~ 0.97; CAS 78-08-0); Acrylic acid (AA solution, CAS 79-10-7); Potassium persulfate (KPS ~ 0.99.99; CAS 7727-21-1); vinyl methacrylate (VMA ~ 0.98; CAS 4245-37-8); Acetic acid (≥ 0.997; CAS 64-19-7). All reagents were purchased from Merck of analytical grade.

### Preparation of native chitosan solution

Add 1.0 gm of chitosan powder to a small amount of pure acetic acid and stir until dissolution. Subsequently, 250 mL of synthetic brine (135,000 ppm) was gradually added under a stirrer at room temperature. The solution was stirred at 500 rpm to prevent mechanical degradation of the chitosan until a homogeneous solution was obtained, according to API-RP-63 guidelines. Finally, the solution was stored in an airtight bottle to prevent evaporation.

### Preparation of composite chitosan solution

**Figure 3:**
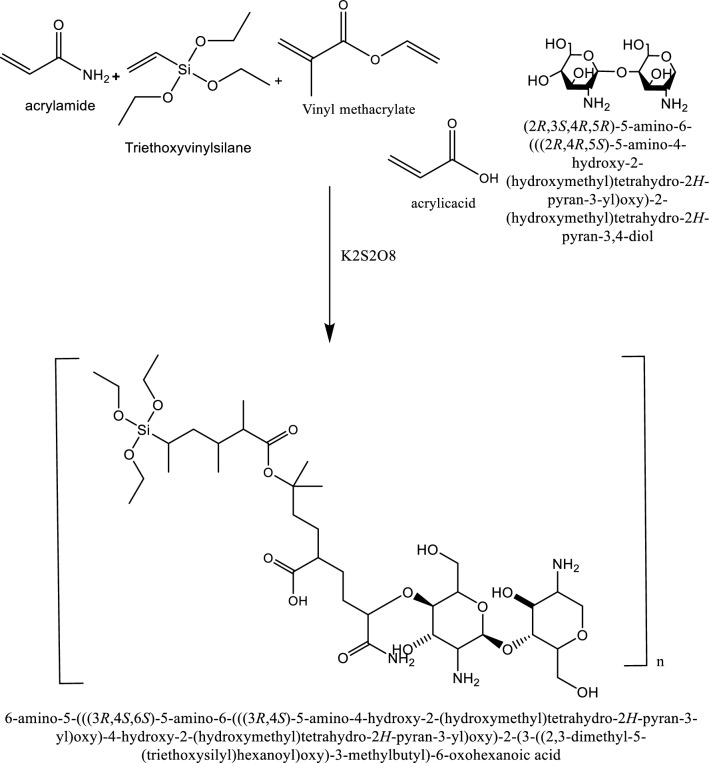
Chemical structure of modified chitosan composite.

0.2 gm of chitosan powder was dissolved in 200 mL of distilled water. Then, 18 gm of AM, 3 gm of CTAB, 3 gm of AA, 1.8 gm of VMA, and 0.2 gm of TEV were added and the solution, and stirred until homogeneity. The resulting mixture was transported to a 3-neck flask and the temperature of the reaction was gradually increased. Oxygen was displaced from the reaction medium by continuously purging with nitrogen gas underwater circulation to diminish evaporation. Once the reaction temperature reached 60 °C, 0.374 gm of KPS initiator was added and the reaction medium was thermally stabilized at 60 °C overnight. The chitosan composite was extracted with acetone, dried, ground into powder, and stored in a desiccator^6^. The chemical structure of the synthesized composite is provided in Fig. 3.

The incorporation or grafting of vinyl monomers, including acrylamide, triethoxyvinylsilane, and vinyl methacrylate onto chitosan plays a crucial role in bolstering the structural resistance of chitosan composites against salts and the hardness of formation water, as, they introduce functional groups that can interact more effectively with the ionic species present in saline waters. This interaction helps in stabilizing the polymer matrix against the osmotic pressures and ionic strengths encountered, thereby maintaining the composite's integrity. Moreover, the incorporation of such vinyl monomers increases the cross-linking density within the chitosan matrix, which not only contributes to its mechanical strength but also reduces its swelling in aqueous environments^37^. Acrylamide, with its high reactivity and crosslinking ability, contributes to the formation of a robust network within the chitosan matrix, enhancing its mechanical strength and durability against saline environments^38^. Triethoxyvinylsilane, on the other hand, acts as a coupling agent, facilitating strong adhesion between the chitosan and inorganic surfaces, thereby reinforcing the composite's structural integrity and resistance to water hardness^39^. Additionally, vinyl methacrylate enhances the composite's chemical stability and provides tailored functionality, further fortifying its resilience against salt ions and hardness in formation water^40,41^. Through these synergistic effects, the incorporation of these vinyl monomers into chitosan composites offers a comprehensive solution to mitigate the deleterious effects of salts and water hardness, ensuring prolonged performance and efficacy in various applications, including enhanced oil recovery. The resulting chitosan composites are characterized by FTIR, AFM, TGA & NMR analysis. The rheological criteria and geometry of the native and composite chitosan solutions were measured using Anton Paar RheoCpmass^TM^: MCR 102e at reservoir conditions using cone-plate geometry. The shear viscosity of both native and composite chitosan solutions was estimated using a flowability test (steady rate sweep test). Also, the dynamic and viscoelastic properties (storage and loss moduli) were determined using the frequency sweep test. Also, the Herschel-Bulkley model was utilized to describe the viscous flow behavior of all solutions. This model is typically applicable to non-linear fluids with yield stress and is considered precise because it has three adjustable parameters, providing data.

### Core plug displacement

Two core plug crops from the Bahariya formation in Egypt were cut from the formation outcrops and then cleaned and dried in an oven. Table 1 summarizes the basic parameters and dimensions of core plugs. The core bulk volume (V_B_) is calculated as follows:

1 $${\mathbf{V}}_{{\mathbf{B}}} = \, {{\varvec{\uppi}}} \, {\mathbf{r}}^{{\mathbf{2}}} {\mathbf{L}}.$$

**Table 1:** Physical properties and dimensions of core plugs.

Plug no	Plug length (L)	Plug diameter (D)	pore volume (PV)	Bulk volume (BV)	Grain density (GD)	Helheim porosity phi	Air permeability Ka
	(cm)	(cm)	(cc)	(cc)	(g/cc)	(%)	(mD)
1st plug	5.25	3.84	21.82	60.72	2.63	37.2	1304
2nd plug	5.6	3.79	22.74	65.47	2.62	37.8	1818

**Figure 4:**
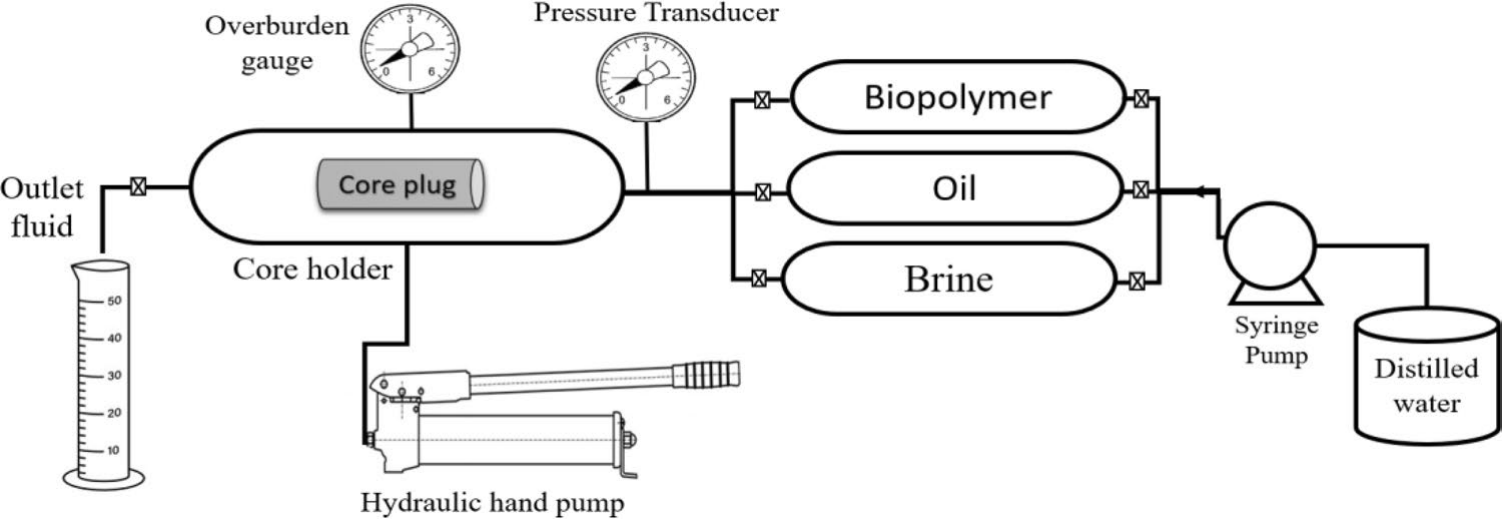
Schematic representation of Core flood system.

The core flood is conducted through the core flooding system as shown in Fig. 4. The core plugs were evacuated before measuring the brine saturation, where the flow rate of the synthetic brine, crude oil, and chitosan (native and composite), were constant during the experiments. The synesthetic brine was displaced to the core plug to determine the absolute permeability. The crude oil (29 API° and 10 cP viscosity at 25 °C) was displaced to determine initial water saturation, oil permeability, and the original oil in place (OOIP)^42^. OOIP can be calculated by flooding the crude oil in the brine-saturated core till no brine is ejected in the outlet effluents while the following formula can be used to determine the initial water saturation:

2 $$S_{wi} = \, \left( {V_{P} - W_{P} } \right)/V_{P} ,$$

where Wp is the produced water calculated from the outlet effluents. Different pore volumes of the synesthetic brine were flooded (0.25, 0.5, 0.75, 1, 5, 10 & 15 of PV) to calculate residual oil saturation, (S_or_). The oil outlet, water ejected, time, and pressure difference ∆p are measured at each injected pore volume. The cumulative produced oil (N_P_) from water flooding and the S_or_ can be calculated as follows:

3 $$Sor \, = \, \left( {OOIP - N_{P} } \right)/ \, V_{P} .$$

To estimate the residual oil saturation after polymer flooding (S_orp_), the chitosan (native/composite) solution is inoculated into the core plug, where the ejected oil volume and the total oil produced (N_PT_) from water displacement and polymer flooding are calculated. Then the S_orp_ can be calculated as follows:

4 $$Sorp = \, \left( {OOIP - N_{PT} } \right)/V_{P} .$$

Owing to the adsorption of polymer on the rock surface which leads to a variation of core physical properties, the resistance factor (R_F_) and the permeability reduction factor (R_K_) are estimated. The R_F_ is the product of dividing the pressure differences during biopolymer (**∆P**_**p**_**)** & water flooding (**∆P**_**w**_**)**. While R_K_ represents the ratio of the brine effective permeability to the biopolymer effective permeability. The R_F_ and R_K_ can be calculated by the following formulae^43^.

5 $${\mathbf{R}}_{{\mathbf{f}}} = \, \Delta {\mathbf{P}}_{{\mathbf{p}}} /\Delta {\mathbf{P}}_{{\mathbf{w}}} ,$$

6 $${\mathbf{R}}_{{\mathbf{k}}} = \, {\mathbf{K}}_{{\mathbf{w}}} / \, {\mathbf{K}}_{{\mathbf{p}}} .$$

### Development of simulation model

The obtained data from core flooding experiments included concentrations of synthetic brine (135,000 ppm), chitosan solution (4000 ppm), chitosan composite solution (1000 ppm), and crude oil gravity (29°API) from Bahariya formation. Furthermore, the rheological and core flooding data are introduced into the tNavigator simulator to be simulated on a reservoir scale to detect changes in water viscosity, and mobility ratios with time and to mimic those changes in different locations throughout the reservoir and between injectors and the producer well sites.

## Discussion and results

### Chitosan composites characterization

**Figure 5:**
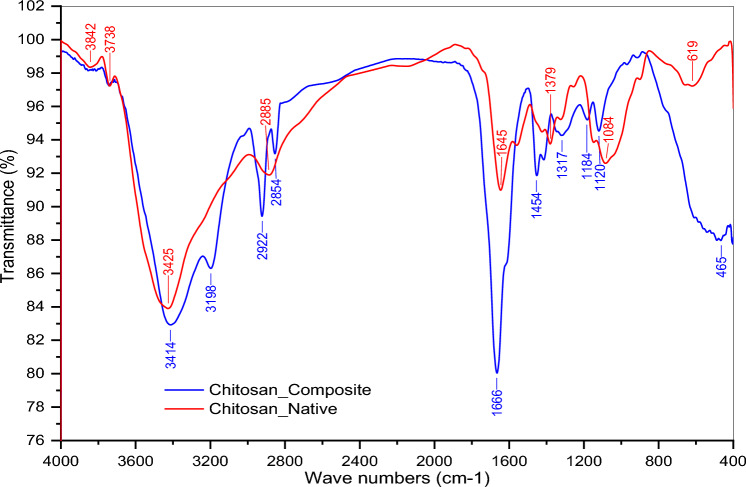
FTIR of native and composite chitosan.

**Figure 6:**
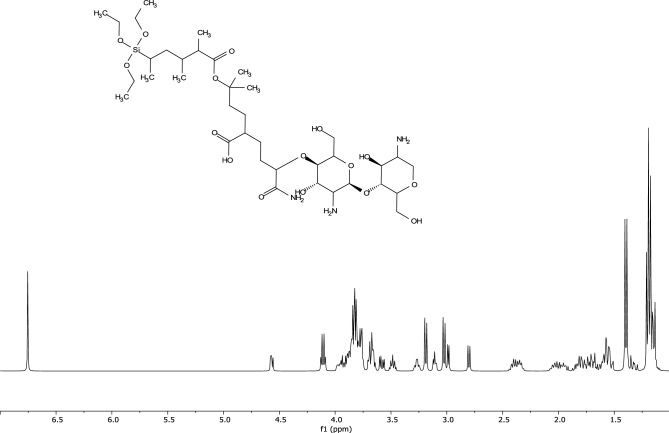
Proton ^1^H NMR spectra for chitosan composite.

The FTIR spectra and proton chemical shift ^1^H-NMR of native and composite chitosan are summarized in Table 2, and displayed in Figs. 5 and 6 respectively.

**Table 2:** FT-IR spectra and chemical shifts of native xanthan and modified composite.

	Frequency (υ, cm^–1^)	Peak justification
FT-IR spectra		
Native chitosan	3842, 3738	Stretching vibration of –OH group
	3425	Stretching vibration of –NH_2_ group
	2885	Stretching vibration of –CH groups in methyl &methylene groups
	1645	Bending vibration of NH primary amine^44^
	1379, 1084	Allocated to bending of C–H side chain in CH_2_OH & C–O glucose bending respectively
	863	C–N stretching vibration
Chitosan composite	3414	The broadband corresponding to the overlapped stretching vibration (–NH_2_) group in acrylamide & chitosan
	3198, 2922	Stretching vibration of –CH groups in grafted vinyl monomers
	1666	Broadband corresponding to stretching vibration of –C=O groups in AM, AA, and VMA monomers
	1454, 1120—465	υ_(Si–O–Si)_ stretching and bending vibrations of (Si–O–Si) bond in triethoxyvinylsilane^45^

	Chemical shift (δ, ppm)	Peak identification
Proton chemical shifts (^1^H-NMR)^46^		
Chitosan composite	δ = 1.17–1.7	(m, 9H, ((Si–O–CH_2_–**CH**_**3**_)) of triethoxyvinylsilane
	δ = 1.58–1.7	(m, H, an aliphatic chain of methylene groups other than chitosan
	δ = 3.03	(t, 2H, (NH_2_–C–OH) of chitosan
	δ = 4.01	(t, H, (**OH**–CH_2_–)) terminal hydroxyl on chitosan
	δ = 6.75	(s, 2H, (CO**NH**_**2**_–) of acrylamide;

Significant values are in bold.

**Figure 7:**
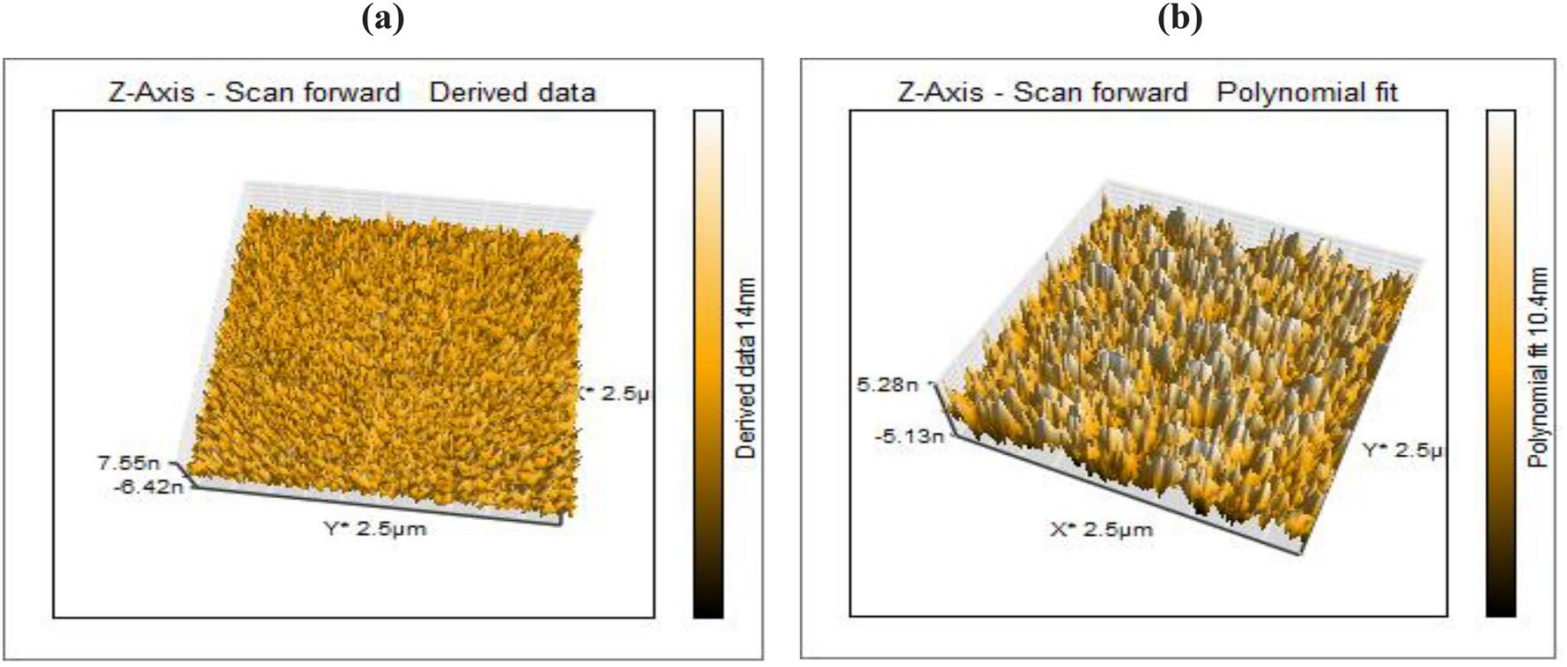
AFM analysis for (**a**) the native and (**b**) composite chitosan.

To acquire high-resolution elevation maps or topographic images of the surfaces at an atomic scale resolution for both native and composite chitosan the atomic force microscopy (AFM) analysis was used^47,48^. As displayed in Fig. 7, the AFM images demonstrate differences in the surface properties of the native and composite chitosan. In Fig. 7a, the surface of native chitosan displays a uniform and smooth appearance due to the presence of interconnected and multilayered fibers that form entangled and diffused networks. This is in contrast to the chitosan composite, which exhibits a different surface morphology. This topography confirms the homogeneous morphology and distribution features of a discrete, elongated, irregular granular structure^49–51^. The chitosan composite surface (Fig. 7b) displays an irregular, globular topology containing protrusions and voids within its interconnected fibers compared to the smooth surface of native chitosan. This topography confirms the grafting and embedding of vinyl monomers within the chitosan surface^50,52^.

**Figure 8:**
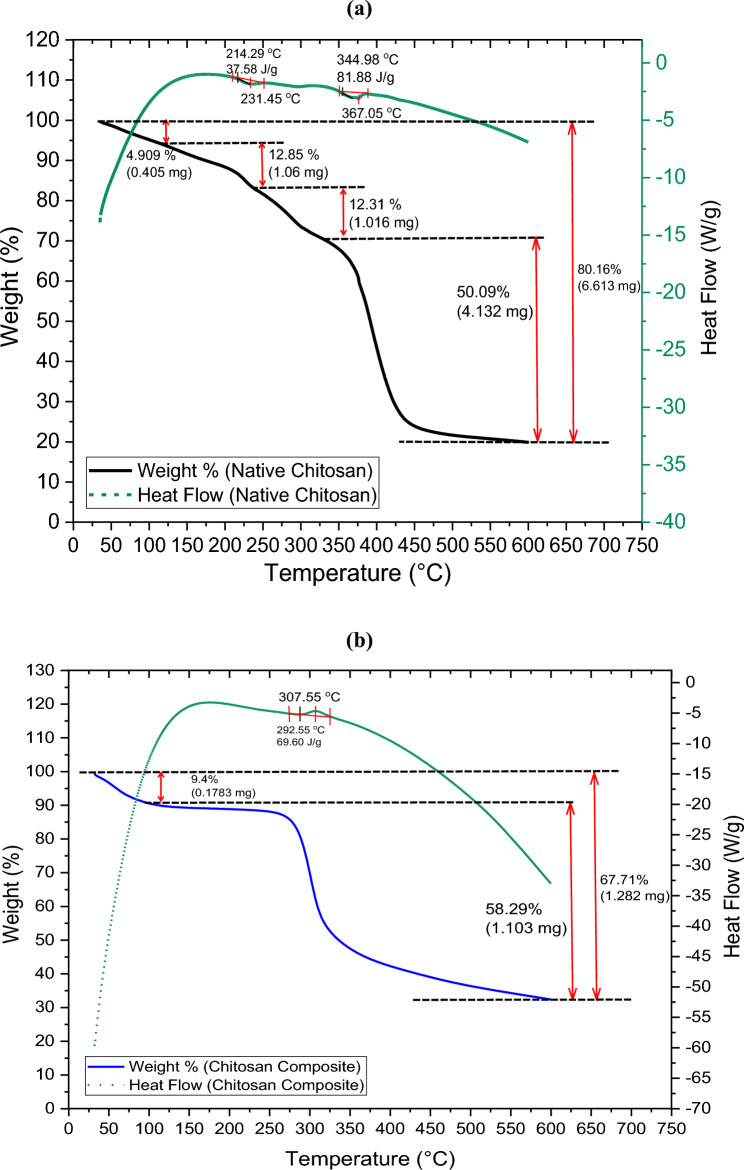
TGA Analysis of (**a**) native and; (**b**) composite chitosan.

The thermal stability of native and composite chitosan was investigated using thermal gravimetric analysis (TGA). The native chitosan (Fig. 8a) goes through four stages of weight loss: 4.9% weight loss at 105 °C due to water evaporation, 12.85% between 105 and 246 °C due to biopolymer chain degradation, 12.5% between 246 and 336 °C due to the loss of side chains, small molecules, and decomposition of chitosan backbone^53^. The remaining weight reaches 20 Wt% after complete decomposition. The chitosan composite (Fig. 8b) shows two decomposition stages: moisture loss (9.4%) between 30 to 110 °C and pyrolysis of polysaccharides (58.29%) from 110 to 350 °C, followed by complete pyrolysis above 350 °C. The chitosan composite has a higher initial decomposition temperature and a second decomposition stage shifted to 450 °C, where the remaining weight % reaches 33% after complete pyrolysis, indicating improved thermal stability due to cross-linking and grafting of silane-containing vinyl monomers^34,54^.

Differential scanning calorimetry (DSC) thermograms provide valuable insights into the thermal properties and transitions of materials where endothermic and exothermic peaks indicate specific thermal events. The DSC thermogram of the native chitosan shows two endothermic peaks at 231 °C and 367 °C, which corresponds to the dehydration process, where bound and structural water molecules are released at lower temperatures and is a characteristic feature of polysaccharides like chitosan. The other endothermic peak at higher temperatures corresponds to the thermal decomposition or depolymerization of chitosan and signifies the breakdown of the polymer backbone into smaller units. The exothermic peaks at 214 °C and 344 °C correspond to the crystallization or reorganization of the polymer chains as they cool and settle into a more stable, ordered structure after melting^55,56^. The DSC thermogram of chitosan composite displays an endothermic peak at 292 °C representing the dehydration of chitosan composite and the thermal decomposition of its polymeric chains. The presence of silane-containing vinyl monomers enhances the thermal stability of the composite by acting as thermal barriers, which might shift the decomposition temperature to higher values or alter the intensity of the endothermic peaks compared with native chitosan. The exothermic peak at 307 °C, is related to the crystallization or reordering of chitosan/ silica cross-linking upon cooling^54,57,58^.

### Rheology of the displacing fluids

**Figure 9:**
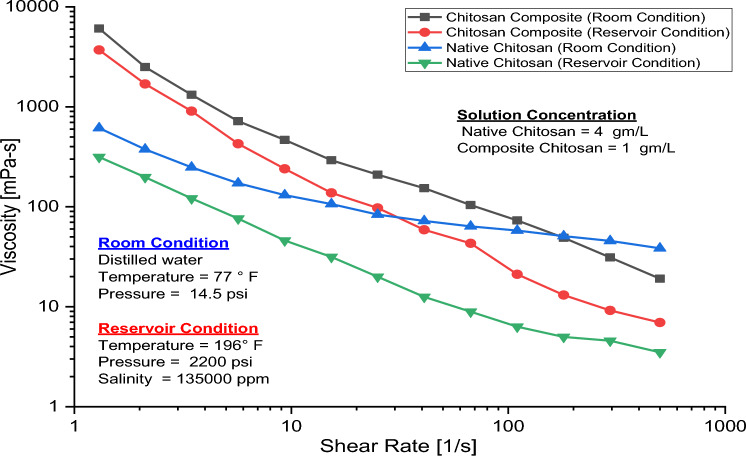
Viscosity-shearing effect for native and composite Chitosan.

The term rheology is defined as the science of the deformation and flow of different fluids as a result of applied stress or strain on them^59^. The viscosity curves for native and composite chitosan at room and reservoir temperatures as shown in Fig. 9 indicate that viscosity decreases at higher shear rates since the biopolymer chain molecules cannot connect or entangle with each other easily under these conditions. Instead, the chains are stretched by the flow, leading to unraveling and disentanglement of the biopolymer molecules. As the shear rate increases, the viscosity of these polymer solutions declines, resulting in shear-thinning behavior^34,60^. By pressure increasing, the internal free volume of the biopolymer structure decreases, so the viscosity increases owing to structure compactness, and limitation of the molecules' free mobility. This limitation in the molecule’s mobility increases the internal friction force and as a consequence the flow resistance^61^*.* On the other hand, the increase in temperature and salinity reduces the solution's viscosity. As the temperature increases, the free volume inside the biopolymer structure increases, so the units become less constrained, more active, and less organized, and their relaxation times are reduced, which results in viscosity reduction^62^. By increasing the solution salinity, the ionic charge of the biopolymer macromolecular structure is neutralized by the brine cations. Hence, the elongated chitosan molecules transform into a helical molecular shape that occupies a smaller volume, resulting in viscosity reduction^15,63,64^*.* The negative effects of both temperature and salinity on the solution viscosity were less severe on the chitosan composite solution than on native chitosan, due to the presence of silane nanoparticles which intensify the structure compactness and rigidity through the formation of interconnected 3D-network^65,66^. Furthermore, nanoparticles shield the chitosan macromolecular structure from brine cations attack through electrostatic charge stimulation, thus strengthening the polymer structure^64,67^*.*

**Figure 10:**
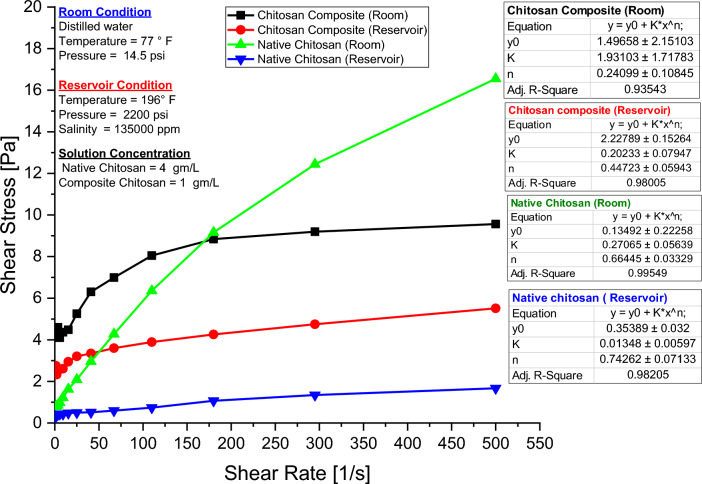
Shear rate versus shear stress for native and composite chitosan.

Figure 10 shows that the Herschel-Bulkley model, with the following equation, provides a good fit to the experimental data presented in Table 3 for both native & composite chitosan samples, with minimal standard errors:

7 $$\tau = \tau_{0} + K*\gamma^{n} ,$$

where τ is shear stress, γ is shear rate, K is consistency index, τ_0_ is yield stress, and n is flow behavior index. The n-value of both native and composite chitosan is less than unity as shown in Table 3, indicating that they are pseudoplastic fluids and exhibit shear-thinning properties. An increase in shear rate results in a decrease in molecular entanglement, leading to a reduction in crosslinked points and subsequently decreasing flow resistance in the system^65^, which reduces the solution viscosity during biopolymer displacement operations through porous media^34,68–70^. The presence of yield stress indicates that the polymer does not undergo any substantial flow below a particular stress threshold. This implies that a three-dimensional network has formed within the hydrogels due to crosslinking and hydrogen bonding, even without the application of shear forces^71,72^.

**Table 3:** Herschel Bulkley model parameters for native and composite chitosan.

Chitosan type	Experiment condition	Yield point, y_0_	Consistency index, (k)	Flow behavior index, (n)	Adjusted R-square
Composite	Ambient condition	1.496	1.931	0.2401	0.935
	Reservoir condition	2.228	0.202	0.447	0.980
Native	Ambient condition	0.315	0.271	0.664	0.995
	Reservoir condition	0.354	0.015	0.743	0.982

**Figure 11:**
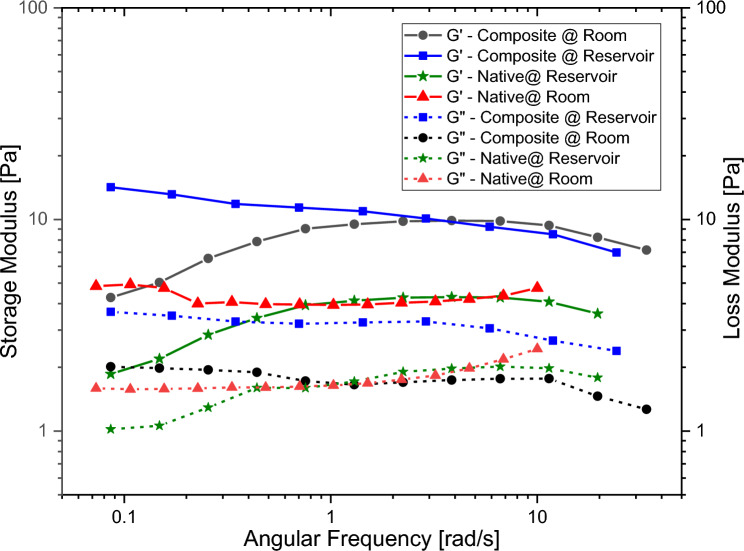
Chitosan native & composite storage/loss modulus.

The frequency sweep test measured the variation of the storage (G‵) and loss (G‶) moduli of native and composite chitosan solutions with the applied angular frequency as shown in Fig. 11. The G‵ modulus was consistently higher than the G‶ modulus for both solutions, indicating viscoelastic solid behavior that increased with frequency^73^. The higher the G‵ modulus, the greater the cross-linking degree and the higher the ability to elastically store energy. Since the loss factor was less than 1.0 for both solutions, they acted as viscoelastic gels. Solutions with G‵ modulus over 10 Pa were strong gels that stored more energy. The storage modulus decreased with increasing temperature, likely due to weakening non-chemical cross-links. This leads to a reduction in intermolecular forces, causing the hydrogel to shift from elastic flow to plastic flow behavior^61,74–76^.

**Figure 12:**
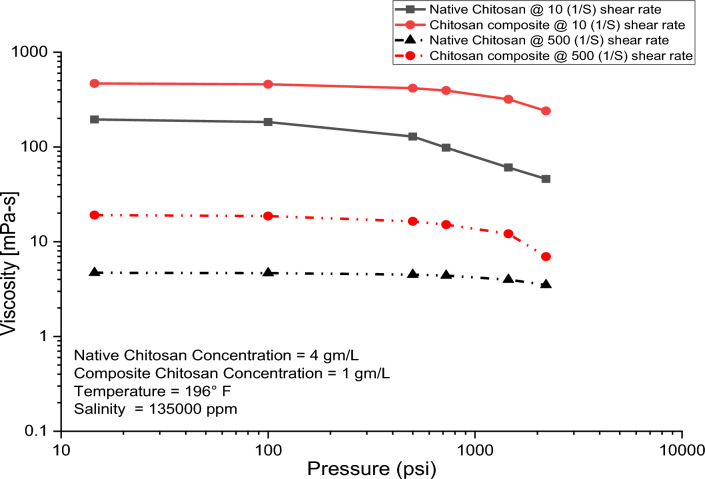
The pressure effect on the native and composite chitosan viscosity.

Figure 12 illustrates the impact of increasing pressure on the viscosity of native and composite chitosan. As pressure rises, the viscosity of both solutions reduces. However, for composite chitosan, the viscosity remains relatively constant until 750 psi before diminishes, whereas for native chitosan, viscosity begins to decrease immediately with increasing pressure. Moreover, the deformation rate for native chitosan surpasses that of composite chitosan after 750 psi. Numerous factors contribute to the reduction in viscosity when pressure rises. One of these factors is that chitosan solutions contain long polymer chains that are entangled with each other. As pressure increases, the polymer chains compress, reducing the entanglements and interactions between the chains, leading to weaker intermolecular forces and lower resistance to flow, so the solution viscosity decreases, due to a transition of chitosan chains from helical to random coil forms under pressure force^77,78^. Furthermore, chitosan chains are linked together by hydrogen bonds between hydroxyl and amide groups, so high pressure can break some of these hydrogen bonds, leading to the weakening of the network structure and lowering the viscosity^79^.

**Figure 13:**
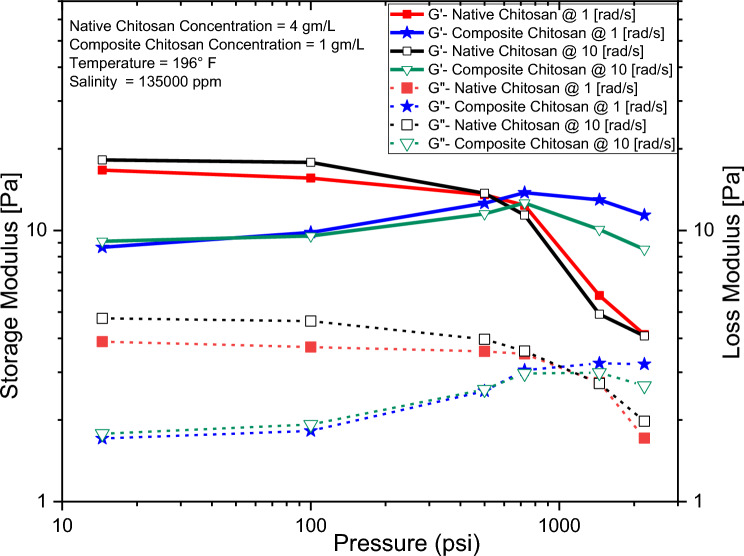
The pressure effect on the storage & loss moduli for native and composite chitosan at a different angular frequency.

The pressure effect on the (G‵) and (G‶) moduli which describe the viscoelastic behavior of chitosan solutions are shown in Fig. 13. It has been observed that increasing pressure can increase the loss modulus and decrease the storage modulus of chitosan solutions. This behavior resorts to the compression of the polymer chains in the solution. As pressure increases, the polymer chains become more compacted, leading to an increase in energy dissipation and a reduction in energy storage. At low pressures, these interactions are weak, and the polymer chains can move more freely, resulting in a lower loss modulus and higher storage modulus^80^. However, at high pressures, these interactions become stronger, causing the polymer chains to become more rigid and less able to store energy. Overall, the increase in loss modulus and decrease in storage modulus with increasing pressure can be attributed to both the compression of polymer chains and the strengthening of intermolecular interactions^15,60,81–84^.

### Core flooding results

Core flooding is initiated by water displacement with several pore volumes to determine the maximum recovered oil through water flooding^66,85^. After that, half the pore volume of the chitosan solution was inoculated, followed by water injection to evaluate the extra oil recovery. The second run was conducted through the chitosan composite to analyze and compare the performance of native and composite chitosan. The initial oil in place for both plugs is 15 cc and the initial water saturation for the first and second plugs was 31.24% and 34.02 respectively. The core flooding data for native and composite chitosan are summarized in Table 4.

**Table 4:** Results of the core plugs flooding.

Polymer solution	Plug	Kw@100% Sw	Ko@Swi, mD	Brine flooding			Biopolymer flooding			Recovery factor, %		Biopolymer resistance factor (RF)	Biopolymer permeability reduction factor (Rk)
				Sor, %	∆P_w_, psi	Kw@Sor, mD	S_orp_, %	∆P_p_, psi	Kwp@S_orp_, mD	2nd recovery	3rd recovery		
Native chitosan	1st plug	869	369	21.31	6.9	52.7	18.1	16.3	4.92	69	4.73	5.26	176.74
Composite chitosan	2nd plug	1272	790	30.78	3.2	92.1	25.06	47	6.63	53	8.67	14.69	191.81

**Figure 14:**
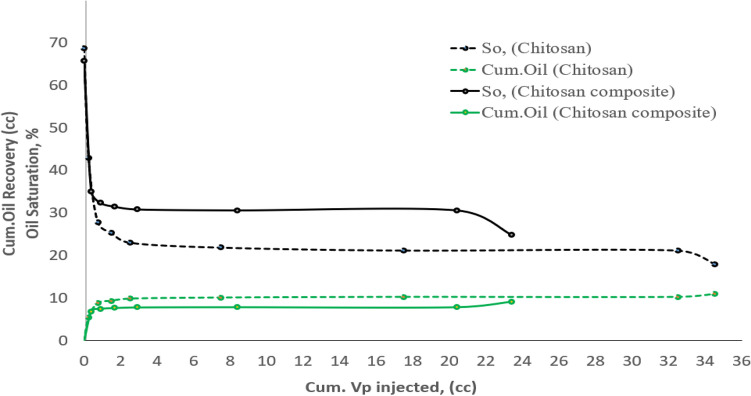
Cumulative oil & saturation profile during plug flooding.

The core flooding results show a variation in pressure differences in both plugs. The results display that in the case of the first plug, the pressure variance between the auto-flood system inlet and outlet was 6.9 psi, compared to 3.2 psi in the case of the second plug during displacement with water. This behavior resorts to the difference in permeability, as shown in Table 3. The results also specify that the recovered oil by water injection from the first plug is much higher than from the second plug, due to the difference in the amount of moveable oil due to the difference in the endpoints. In the first plug, the initial water saturation was 31.24% and the residual oil saturation was 21.31% while in the second plug, the initial water saturation was 34.04% and the residual oil saturation was 30.78%. This means that the moveable oil in the first plug is equal to 47%, compared to 35.2% in the second plug. This is clearly illustrated in the oil saturation and the cumulative oil production as shown in Fig. 14.

After the injection of native and composite chitosan, the recovered oil amounts reveal that the chitosan composite was able to recover 18.6% of the residual oil saturation (Sor), which is equivalent to 8.67% of the plugged oil in place, while the native chitosan was able to recover 15% of the residual oil saturation, which is equivalent to 4.67% of the plugged oil in place. Regarding the effect of chitosan (native and composite) injection on the properties of the rock, the results indicate a permeability reduction owing to the adsorption of a thin layer of polymer debris on the core throats^34^. The first plug permeability decreased from 52.7 to 4.92 mD, while the second plug permeability decreased from 92.1 to 6.63 mD. This led to an increase in the pressure difference in the first plug from 6.9 to 16.3 psi and the second plug from 3.2 to 47 psi. The decrease in plug permeability was reflected directly in the plug resistance factor (R_F_) and permeability reduction factor (R_k_), as shown in Table 4. The flooding results indicate the superiority of the chitosan composite compared to the native one.

## Pilot and field-scale simulation modelling

### Matching of laboratory experiments by tnavigator simulator

**Figure 15:**
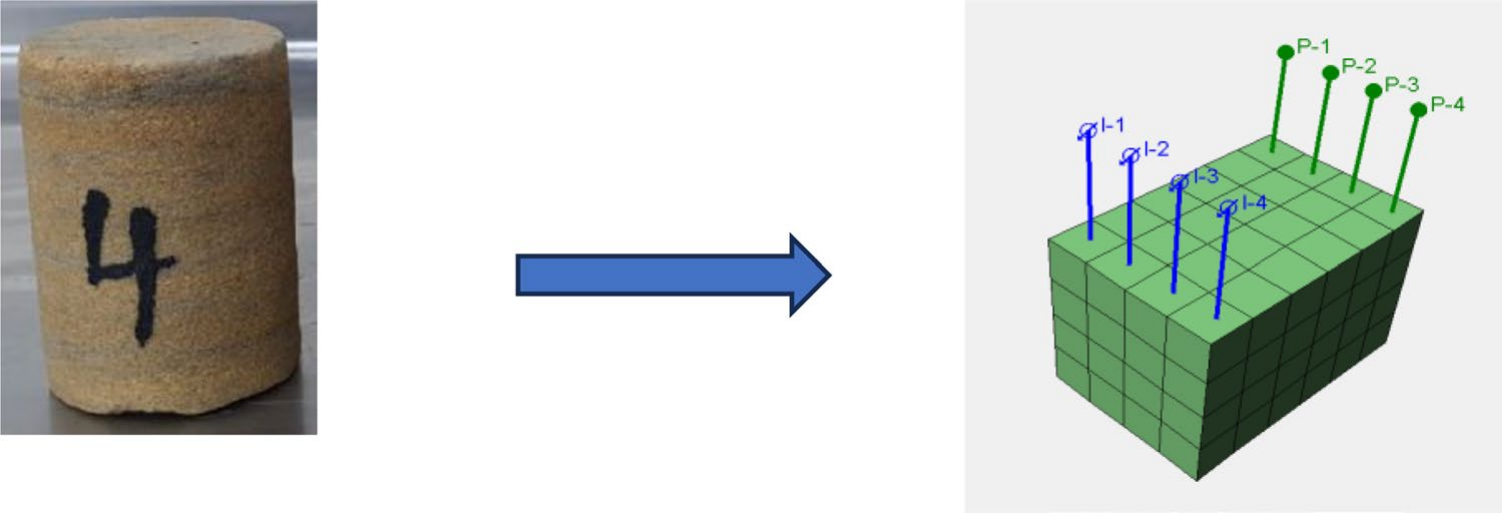
Approximated cylindrical plug to cuboidal rock in the direct line 3D model.

**Figure 16:**
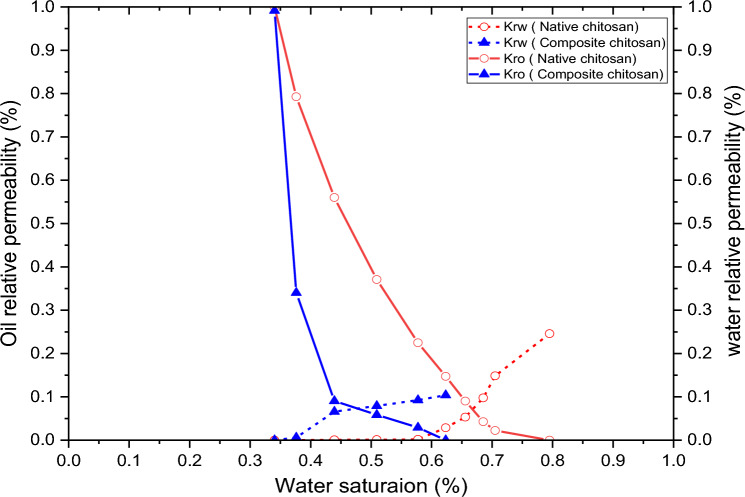
Relative permeabilities curve for native & composite chitosan.

Validation of the experimental results obtained through the core flooding experiments of chitosan (native & composite) through simulation modeling is crucial to overcome certain limitations such as human error, and equipment uncertainty^86,87^. Table 5 shows the input parameter of the simulation model using tNavigator Simulator. A cuboidal rock sample with a dimension of 4 × 4 × 6cm was used to approximate a cylindrical plug in the development of the model as shown in Fig. 15. The model size comprises four producers and four injectors, and the grid size is 1 × 1 × 1 cm. Also, the NX × NY × NZ is 4 × 6 × 4 cm and the total number of the model grid is 96 while the interred relative permeabilities data to the model are shown in Fig. 16 respectively. In this model, a waterflood is performed with 0.9cc/min through the four injectors. Oil recovery and other parameters were calculated at different reservoir pore volumes. The oil recovery, oil saturation, and the additional oil recovery obtained from the simulation model and planned to match with the lab core flood results in each core plug.

**Table 5:** The simulation model inputs for lab and field scale models.

Items		Native chitosan	Composite chitosan
Water viscosity (cp)		0.4	0.4
Oil viscosity (cp)		2	2
Solution viscosity, cp		46	240
PLYVISC	Biopolymer concentration (Ib/stb)	0.789	0.263
	Viscosity multiplier	180	575
PLYROCK	Dead pore space	0.05	0.05
	Resistance factor	5.26	14.69
	Rock mass density (Ib/rb)	800	800
	Adsorption index	1	1
PLYADS	Biopolymer concentration (Ib/stb)	0.789	0.263
	Biopolymer adsorption (Ib/Ib)	0.00005	0.00005
PLMIXPAR	Biopolymer ToddLongstaff parameter	1	1
PLYMAX	Biopolymer concentration (Ib/stb)	0.789	0.263
	Salt concentration in solution (lb/stb)	47	47

**Figure 17:**
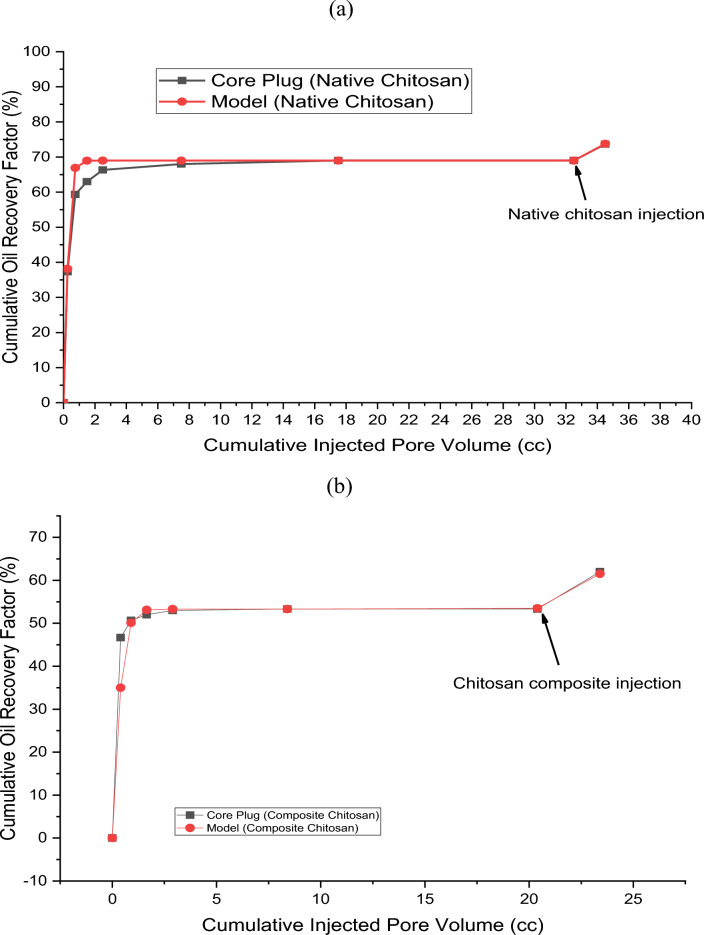
Cumulative oil recovery for core plug & simulation model at different pore volume injections of water and biopolymer. (**a**) Native chitosan; (**b**) Chitosan composite.

Figure 17 illustrates the cumulative oil recovery performance of both the experimental and simulation model data at various levels of pore volume injection. The simulation and experimental data show a higher matching degree. For native chitosan, residual oil saturation was 21.31% after waterflooding, which was similar to the residual oil saturation of 20.61% observed in the simulation model results. The addition of chitosan after waterflooding resulted in the extraction of an additional 3.2% of oil from the reservoir in plug experiments, which was comparable to 3.18% recovery % in the simulation model. Waterflooding was able to recover 69% of oil in plugs and 68.98% in models, while the total recovery after chitosan flooding was 73.67% and 73.77% for core plugs and simulation models respectively. For composite chitosan, plug experiments revealed a residual oil saturation of 30.78% after waterflooding, which was close to the residual oil saturation of 30.09% observed in the simulation model. Additionally, composite chitosan flooding after waterflooding was able to extract an additional 5.72% of oil in plugs, which was higher than the 5.17% increase observed in models. Waterflooding was able to recover 53.33% of oil in plugs and 53.50% in models, while the total recovery after composite chitosan flooding was 62% for plugs and 61.52% for models. These data reflect a consistency between the laboratory and model results. There is a slight deviation at the beginning of the chitosan composite injection, but it does not significantly affect the final recovery values. The percentage of average deviation error between the experimental and simulation model results is 3.42% for native chitosan and 10% for composite chitosan. These deviations are within the acceptable range of modeling and can be attributed to variations in pore volume between the actual core and the modeled plug.

### Oil field case simulations

#### Model description

**Figure 18:**
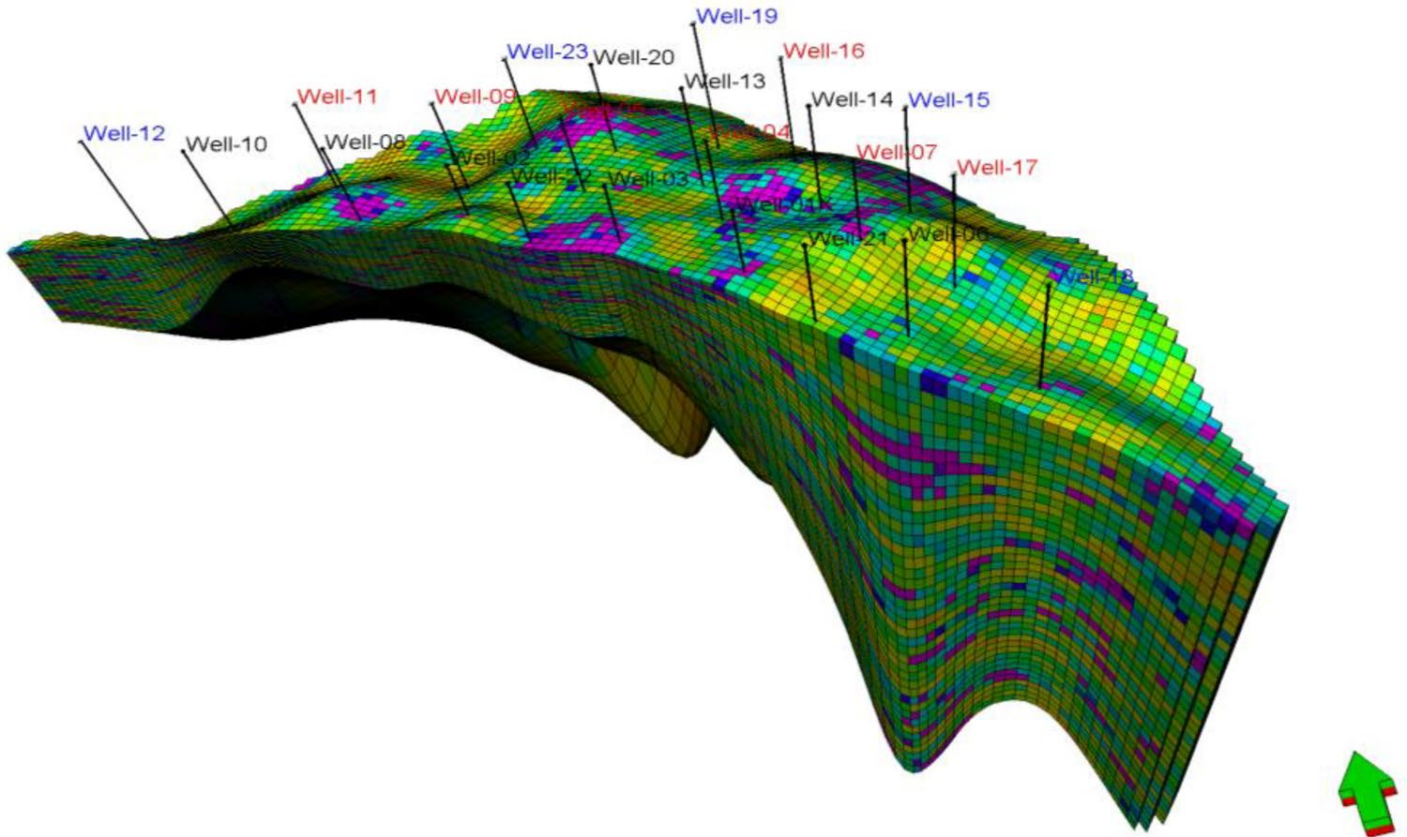
The studied reservoir 3D model.

The assigned field is located in the western desert and consists of 23 wells (11 producers and 12 injectors) as shown in Fig. 18. The producing reservoir is the Bahariya Formation, located in Egypt. This reservoir is working under a depletion drive mechanism and is planned to use the water injection from day one and the biopolymer in the secondary stage. A direct line pattern injection will be used to ensure the highest oil recovery. The reservoir produced oil gravity is 29° API, viscosity of 10 cp at ambient conditions, and was located at a 6500 ft depth. The reservoir pressure and temperature are 2600 psi and 195 °F respectively. The model size is 1200ft × 1760 ft × 19ft, the grid size is 160ft × 160ft × 6ft, NX × NY × NZ is 117 × 39x40 and the total number of the model grid is 182,520 while the interred relative permeabilities data to the model are shown in Fig. 16 respectively. The other required model parameter is inferred from Table 5. In this model, 7250 barrels of water per day was injected through the 12 injectors while 7000 barrels of oil per day was produced through 11 producers. The reservoir performances and the changes in the water viscosity, polymer concentration & mobility ratio with time at different locations in the whole reservoir and between the injectors and producers are simulated.

**Figure 19:**
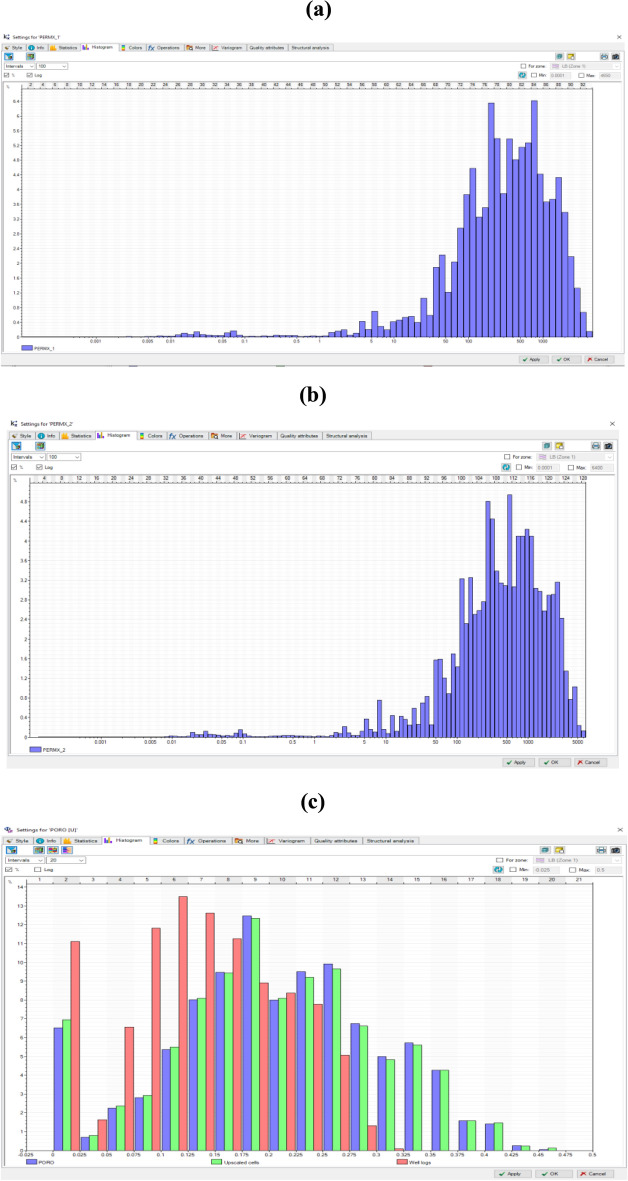
Permeability and porosity of (**a**) Native chitosan; (**b**) Composite chitosan (**c**) histogram of the entire 3D model.

The permeability and porosity distribution entire the 3D geological model is displayed in the histogram shown in Fig. 19. The permeability ranged from (10–4000 md) and from (5–5000 md) for the native and composite respectively, while the porosity range, is almost the same (0.1–0.35) for both native and composite biopolymer models.

#### Field model simulation results

**Figure 20:**
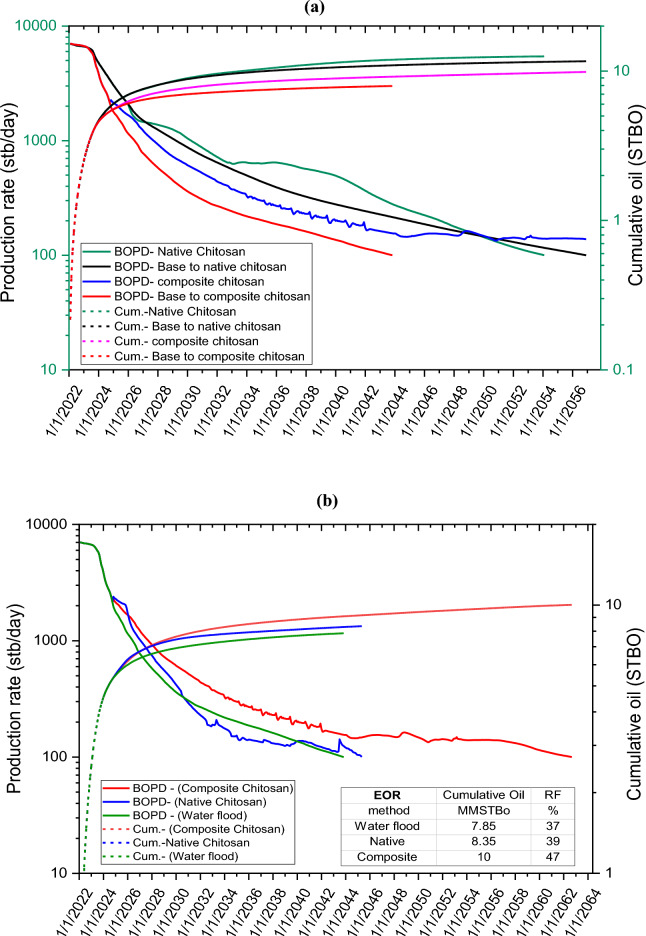
Oil production rate and cumulative recovery for native and composite chitosan. (**a**) Different reservoir quality, (**b**) same reservoir quality.

The efficacy of water displacement, and native, and composite chitosan flooding in enhancing oil recovery was evaluated through this model. The base case involves the use of water flood, and its performance will be compared to the performance of native and composite chitosan flooding. The field production performance will be monitored until it reaches the economic limit in all cases. For the native and composite chitosan cases, half pore volume will be injected when the water percentage reaches 70%, based on a previous optimization study. This will be followed by water injection until the economic limit is reached. The simulation results for each case are presented in Fig. 20a and Table 6, which demonstrate the superiority of composite chitosan over water and native chitosan. The results indicate that the composite was able to add 10% recovery % more than water flooding compared with 4% oil recovery for native chitosan. Additionally, the difference in the total cumulative oil values in Table 6 is attributed to the disparity in the plug endpoints in the case of native and composite chitosan. In addition, Fig. 20b illustrates the production performance of water flooding, and native and composite chitosan flooding when injected into a single reservoir with the same rock quality and endpoints. The high recovery factor achieved by using the chitosan composite in the enhanced oil recovery stage indicates its ability to distinctly displace the reservoir, demonstrating its success and effectiveness in improving the recovery factor of the reservoir.

**Table 6:** Oil recovery factor for water flood, native & composite chitosan at different reservoir quality.

EOR method	Field WC, %	Cumulative oil, (mmstb)	RF, %	Incremental RF, %
Native chitosan	Base case (WF)	11.62	52	4%
	Native	12.53	56	
Composite chitosan	Base case (WF)	7.93	37	10%
	Composite	10.11	47	

**Figure 21:**
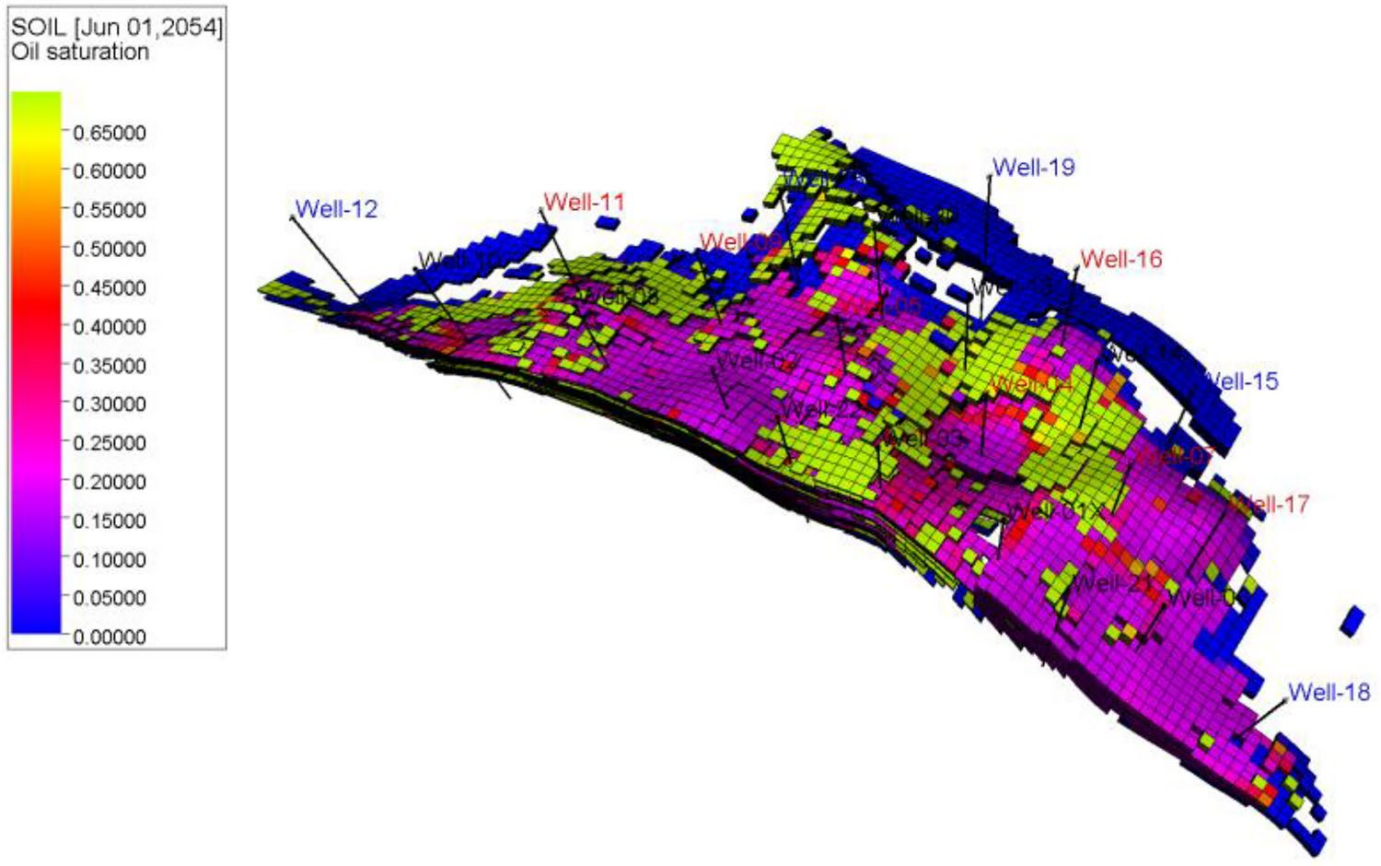
Oil saturation distribution through the entire 3D model after native & composite chitosan flooding.

In Fig. 21, the oil saturation is depicted at the point where the economic oil limit is reached. In most cases, the oil saturation at the end of the reservoir's production life does not exceed 25%. However, certain isolated streams or very tight areas in the reservoir may contain oil with higher saturation levels of around 65% or slightly above.

**Figure 22:**
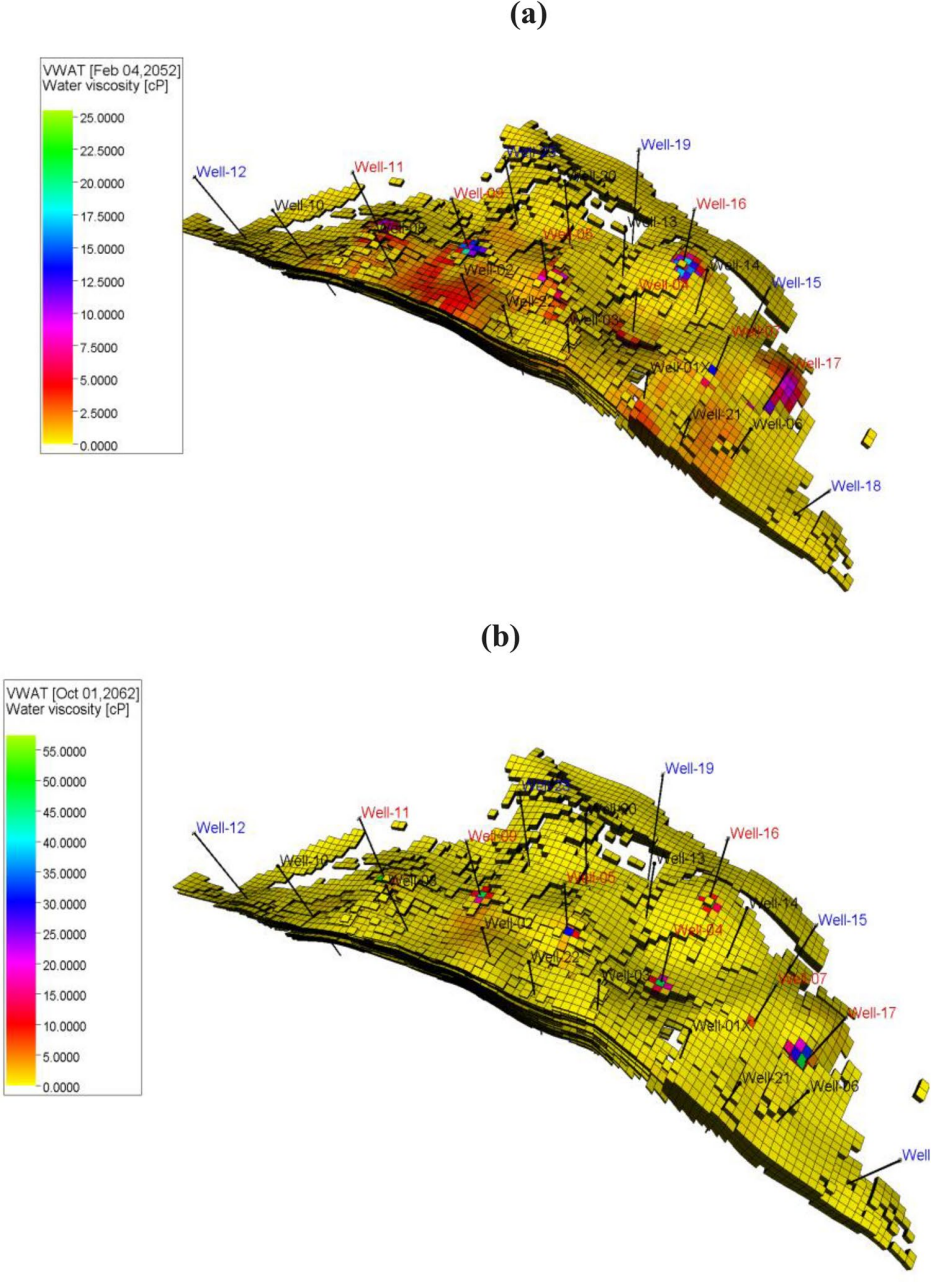
Water viscosity distribution entire 3D geological model after native, and composite chitosan flooding. (**a**) Native chitosan, (**b**) Composite chitosan.

The water viscosity distribution of entire the 3D geological model at the economic oil limit is displayed in Fig. 22. The study reveals that for native chitosan (Fig. 22a), there were numerous areas in the reservoir where water viscosity increased from 0.5 to 5 cp, and in some instances, up to 15 cp. This indicates that most of the injected half-pore volume from native chitosan did not reach the producers, but instead entered into tight areas in the reservoir, so became inaccessible. This led to poor sweep efficiency in the reservoir, resulting in a weak recovery rate of only 4% more than the water flooding. Conversely, in composite chitosan (Fig. 22b), only small & limited areas in the reservoir showed an increase in water viscosity to 10 cp at the economic oil limit. This finding suggests that most of the injected composite chitosan was produced through producers, indicating an improvement in reservoir sweep efficiency and an increase in the recovery rate, which reached 10%.

## Operational challenges

Based on the core flooding and the presented reservoir model results, the operational challenges in the biopolymer flooding projects can be summarized as follows;

i. Microbial stability: the activity and stability of microbes over long injection periods need to be ensured. Biopolymers can be susceptible to microbial degradation under reservoir conditions. Maintaining the stability and integrity of the biopolymers throughout the flooding process is crucial to ensure their effectiveness in displacing oil^65^.
ii. Reservoir heterogeneity: Biopolymer flooding may face challenges in reservoirs with complex geological formations and heterogeneity. Understanding the reservoir characteristics and designing appropriate injection strategies to optimize sweep efficiency is vital for successful biopolymer flooding^88^.
iii. Formation damage: Biopolymer flooding can cause formation damage due to factors such as biopolymer adsorption onto reservoir rock surfaces or the plugging of pore throats. This can reduce reservoir permeability and negatively impact oil recovery. Mitigation strategies, such as pre-flushing the reservoir or using additives to prevent adsorption, may be necessary to minimize formation damage.
iv. Monitoring and Surveillance: Continuous monitoring and surveillance of biopolymer injection operations are essential to assess project performance, detect any operational issues or anomalies, and make timely adjustments. Implementing monitoring techniques such as tracers, surveillance wells, and surface measurements helps optimize reservoir management and maximize oil recovery.

## Conclusion

Modified chitosan through vinyl silylated monomers were prepared and characterized. The results of the rheological assessment revealed that the chitosan composite solution exhibited superior tolerance to salinity, temperature, and pressure even at high shear rates and reservoir conditions relative to native chitosan. The effect of pressure on polymer viscoelastic properties including viscous and elastic moduli was evaluated. The addition of silica nanoparticles to the native chitosan increased the viscosity of the chitosan composite solution by five times. Both native and composite chitosan showed a shear thinning behavior at the reservoir condition of 135,000 ppm salinity, 196°F temperature, and 2200 psi pressure. The core flood experiment on sandstone core plugs indicated that the tertiary stage of flooding with composite chitosan resulted in 8.67% additional oil recovery compared to 4.73% with native chitosan. Moreover, the residual oil saturation decreased by 22% and 15% for composite and native chitosan, respectively, compared to water flooding, which demonstrates the superior sweep efficiency of chitosan composite flooding. Field-scale evaluation using the tNavigator simulator revealed that the composite chitosan recovered 47% of the original oil in place, compared to 39% for native chitosan and 37% for water flooding only in acidic environments.

## Data Availability

The datasets used and/or analyzed during the current study are available from the corresponding author on reasonable request.
